# Outstanding Strengthening and Toughening Behavior of 3D‐Printed Fiber‐Reinforced Composites Designed by Biomimetic Interfacial Heterogeneity

**DOI:** 10.1002/advs.202103561

**Published:** 2021-11-25

**Authors:** Siwon Yu, Yun Hyeong Hwang, Kang Taek Lee, Sang Ouk Kim, Jun Yeon Hwang, Soon Hyung Hong

**Affiliations:** ^1^ Department of Material Science and Engineering Korea Advanced Institute of Science and Technology (KAIST) 291 Daehak‐ro, Yuseong‐gu Daejeon 34141 Republic of Korea; ^2^ Institute of Advanced Composite Materials Korea Institute of Science and Technology (KIST) Jeonbuk 55324 Republic of Korea; ^3^ Department of Mechanical Engineering Korea Advanced Institute of Science and Technology (KAIST) 291 Daehak‐ro, Yuseong‐gu Daejeon 34141 Republic of Korea; ^4^ National Creative Research Initiative Center for Multi‐dimensional Nanoscale Assembly Korea Advanced Institute of Science and Technology (KAIST) 291 Daehak‐ro, Yuseong‐gu Daejeon 34141 Republic of Korea; ^5^ Nanotechnology Research Institute Jiaxing University Jiaxing China

**Keywords:** 3D printing, composites, fiber alignment, hierarchical structures, interfacial heterogeneity

## Abstract

3D printing of fiber‐reinforced composites is expected to be the forefront technology for the next‐generation high‐strength, high‐toughness, and lightweight structural materials. The intrinsic architecture of 3D‐printed composites closely represents biomimetic micro/macrofibril‐like hierarchical structure composed of intermediate filament assembly among the micron‐sized reinforcing fibers, and thus contributes to a novel mechanism to simultaneously improve mechanical properties and structural features. Notably, it is found that an interfacial heterogeneity between numerous inner interfaces in the hierarchical structure enables an exceptional increase in the toughness of composites. The strong interfacial adhesion between the fibers and matrix, with accompanying the inherently weak interfacial adhesion between intermediate filaments and the resultant interfacial voids, provide a close representation of the toughness behavior of natural architectures relying on the localized heterogeneity. Given the critical embedment length of fiber reinforcement, extraordinary improvement has been attained not only in the strength but also in toughness taking advantage of the synergy effect from the aforementioned nature‐inspired features. Indeed, the addition of a small amount of short fiber to the brittle bio‐filaments results in a noticeable increase of more than 200% in the tensile strength and modulus with further elongation increment. This article highlights the inherent structural hierarchy of 3D‐printed composites and the relevant sophisticated mechanism for anomalous mechanical reinforcement.

## Graphical abstract

Here, the authors present that the intrinsic architecture of 3D‐printed composites closely represents biomimetic hierarchical structure composed of intermediate filament assembly among the reinforcing fibers, and thus contribute to a novel mechanism to simultaneously improve mechanical properties and structural features. Notably, the authors find that an interfacial heterogeneity between numerous inner interfaces in the hierarchical structure enables an exceptional increase in the toughness of composites.

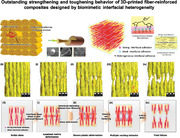

## 1 Introduction

Over the last decades, there have been numerous attempts to emulate or draw inspiration from the natural complex architectures of a biological system that have been optimized by the biological evolution over a prolonged period.^[^ ^1^, ^2^, ^3^ ^]^ Various structural characteristics observed in the biological systems, such as highly ordered fibrillar structures^[^ ^4^, ^5^ ^]^ or 2D planar structures,^[^ ^6^, ^7^ ^]^ as well as fine porous structures,^[^ ^8^, ^9^ ^]^ propose the mechanical requirements for the next‐generation structural materials, while concurrently offering low density, high strength, and high toughness that commonly counterbalance each other. Such meaningful attempts have also been a major motivation for the accelerated advance in 3D printing technology.^[^ ^10^, ^11^, ^12^ ^]^ Recent studies have focused on how to exploit the intrinsic benefits from 3D printing,^[^ ^13^, ^14^ ^]^ that is, a high degree of material orientation and hierarchical multi‐level architecture, as a route toward superior materials with sophisticated structure and composition beyond the pre‐existing manufacturing methods.^[^ ^15^, ^16^, ^17^ ^]^ One example is a natural seashell‐inspired hierarchical architecture built by an electrically assisted 3D printing process.^[^ ^8^, ^12^ ^]^ Seashells are natural nanocomposite armors with an excellent combination of strength and toughness.^[^ ^18^, ^19^, ^20^ ^]^ In this printing process, graphene nanoplatelets are highly aligned by the electric field and act as bricks with the polymer matrix in between as mortar. The resulting robust architecture exhibited natural seashell‐like mechanical prowess beyond conventional expectation.

**Figure 1:**
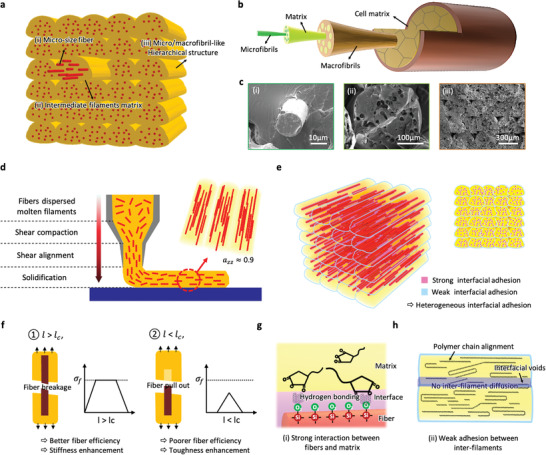
Research strategies for simultaneous improvements of strength, stiffness, and toughness from distinctive architecture by analogy with hierarchically structured nature materials. a) Schematic illustration of 3D‐printed SFRP composites showing fibrous assemblies hierarchically built with individual intermediate filaments, with embedded micron‐sized fibers. b) Detailed illustration of the natural micro/macrofibril hierarchical structure of biological materials. c) Scanning electron microscopy images of the sequential additive architecture of 3D‐printed composites showing structural hierarchy: i) embedded micron fibers, ii) the intermediate filament matrix, and iii) the hierarchical structure. d) Process‐induced spontaneous fiber alignment. 3D printing leads to a high degree of material orientation in the printing direction, especially the alignment of embedded fibers with a high degree of orientation reaching *a*_zz_ = 0.9 (Z direction orientation value). e) Contribution of a heterogeneous interfacial adhesion system to biomimetic toughness behaviors. f) The interfacial adhesion of composites is highly dependent on the critical embedment fiber length; the fiber pull‐out mechanism, which occurs primarily for *l* < *l*_c_, and the fiber breakage mechanism, which occurs primarily for *l* > *l*_c_. Stress‐position profiles show the representative curves when the fiber is ① greater than *l*_c_ and ② less than *l*_c_. *σ*_f_ is the ultimate tensile strength of the fiber. g) Strong interfacial adhesion between the fiber and the matrix was achieved with effective interfacial treatments. h) Weak interfacial adhesion between inter‐filaments spontaneously derived from low inter‐diffusion of polymer chains at the interface.

Our interest here has been focused on industrial materials, especially fiber‐reinforced composites. 3D printing of short fiber‐reinforced plastic (SFRP) composites, which has long been under use in an extensive range of industrial applications, is highly desirable for practically meaningful functional architectures.^[^ ^21^, ^22^, ^23^ ^]^ In general, 3D‐printed SFRP exhibits a multi‐scale hierarchical microstructure, from the micro‐scale embedded fiber architecture to the macro‐scale intermediate filament assembly.^[^ ^24^ ^]^ Intriguing hierarchical structures can be realized, consisting of intermediate filaments well‐aligned in the printing direction and impregnated with highly oriented reinforcing short fibers^[^ ^25^, ^26^ ^]^ (**Figure** **1**a). This structural feature commonly results in highly anisotropic mechanical behaviors along with a dramatic increase in the strength along the preferred direction.^[^ ^27^, ^28^ ^]^ As such, 3D printed fiber‐reinforced composites offer a distinctive architecture uniquely driven by 3D printing, which can also be judiciously understood based upon the inspiration from natural biological systems. For example, we are well aware that natural bamboo has anisotropic mechanical behavior. Bamboo fractures typically follow specific patterns, and fiber orientation in bamboo has a significant effect on crack development. 3D printing has been used to represent such a sophisticated structure, and explore the structure–property relationships in bioinspired and biological materials.^[^ ^29^, ^30^ ^]^

As aforementioned, most studies have recognized the high degree of orientation of reinforcing materials, which are guided during the 3D printing process, as being positive in terms of strength reinforcement;^[^ ^31^, ^32^ ^]^ in our previous works, the fiber alignment in various printing conditions, such as print pattern and fiber concentration, has been thoroughly investigated and comprehensive comparisons with the conventional engineering composites in terms of microstructure and mechanical properties have been addressed as well.^[^ ^33^, ^34^ ^]^ Our results demonstrated that precise fiber adjustment of 3D printing fabricates sophisticated microstructures and determines correlating mechanical strength.

In contrast, the spontaneous formation of imperfect interfacial voids has been viewed as a disadvantage.^[^ ^34^, ^35^, ^36^ ^]^ Much attention has focused on an inevitable void formation and consequent development of crack growth path and interfacial delamination, a major factor limiting the performance of thermoplastic extrusion 3D printing.^[^ ^37^, ^38^, ^39^, ^40^ ^]^ However, controlled void formation may not be a limitation in terms of nature‐inspired engineering, although often times considered undesirable. Recent studies have shown biomimetic composites being printed with engineered voids. By strategically incorporating voids into the load paths, complex fracture behavior may be realized, wherein the effects of crack deflection and crack blunting are implemented in an engineered manner.^[^ ^41^ ^]^

Noteworthy, this interesting approach for complex composite structures raises the critical issue in the contact interfaces among the different structural elements, such as fiber‐to‐matrix, or filament‐to‐filament interfaces.^[^ ^42^ ^]^ To date, the scientific community has principally focused on the structural construction of these types of composites,^[^ ^43^, ^44^ ^]^ while overlooking the in‐depth study of the interfaces.^[^ ^45^ ^]^ In this work, we present a distinctive synergistic mechanism for the mechanical property enhancement in 3D‐printed hierarchical fiber‐reinforced composites with an interfacial heterogeneity. The intrinsic architecture, including highly aligned fibers as well as interfacial voids, can synergistically serve in a similar way with the micro/macrofibril‐like hierarchical structures in a natural biological system to yield the unique strength and toughness reinforcements (Figure 1b,c). Notably, the interfacial heterogeneity between numerous inner interfaces in the hierarchical structure enables an anomalous increase in the toughness of composites. Indeed, the addition of a small amount of short fiber to the brittle bio‐filaments resulted in a noticeable increase not only in the tensile strength and modulus (by more than twofold) but also in the elongation rate and, consequently, simultaneous enhancement of the strength, stiffness, and toughness was attained. Nature has evolved a structural architecture with heterogeneous media to improve fracture toughness in contrast with man‐made materials with uniform microstructure and homogeneous properties.^[^ ^46^ ^]^ The structural features of natural materials here can be linked to the optimized architecture configuration, the thorough arrangement of constituent elements in a hierarchical manner, and the delicate balance between weak and strong interfacial interactions among such elements based on the heterogeneous nature.

## 2 Results and Discussion

Figure 1 shows the remarkable approaches (i)–(iii) in this study.

i) 3D printing of fiber‐reinforced composites that exhibit natural hierarchical architecture (Figure 1a–d). 3D printing induces a high degree of material orientation in the printing direction to form a series of fibrous assemblies hierarchically built with individual intermediate filaments with embedded micron‐sized fibers. This anisotropic architecture is attributed to a shear‐driven printing process and depends on the viscoelastic behaviors of the composites in the molten state.
ii) Contribution of interfacial interactions for the close representation of toughness behaviors of natural architectures, which depend on the localized heterogeneity (Figure 1e). Notably, an intimate interfacial adhesion among the fibers and matrix within intermediate filaments, accompanied by inherently imperfect interfaces, including weak interfacial adhesion and resultant interfacial voids among intermediate filaments, closely represent a heterogeneous interfacial system. This interfacial heterogeneity may trigger a biomimetic toughness behavior. While strong fiber‐matrix adhesion can be controlled with an interfacial treatment (Figure 1g) (e.g., inter‐molecular hydrogen bonding between induced polar functional groups), weak adhesion among the intermediate filaments is relying on the low inter‐diffusion of polymer chains highly aligned in the printing direction. (This weak interface eventually can develop into interfacial voids between inter‐filaments) (Figure 1h).
iii) Discontinuous fiber reinforcements with an effective length (Figure 1f). Continuous fiber consumes a significant amount of ductility in composites. Short fiber‐reinforced polymer composites, by contrast, satisfy our proposed strategy, while synergistically integrating the strong fiber strength with the toughness of the polymer matrix. The effective fiber length, which represents the embedment fiber length (*l*) longer than the critical embedment fiber length (*l*_c_), is one of the critical parameters to achieve the desired strength enhancement.^[^ ^47^, ^48^ ^]^ The effective fiber length determines the efficiency of load transfer from the fiber to the matrix and drives an unusual reinforcement mechanism from toughness to stiffness or vice versa. In addition, the interfacial adhesion between the fiber and matrix is highly dependent on the *l*_c_ of embedded fibers. *l*_c_ correlates with the interfacial properties, as follows:
(1) $$l_{c}=\;\frac{\sigma_{f}\gamma }{\tau }$$

where *σ*_f_ is the strength of fiber, *r* is the radius of the fiber, and *τ* is the interfacial shear strength (IFSS) between the fiber and the matrix.

As noted above, all these features are commonly used to represent the natural hierarchical structure and serve to design a toughness reinforcement mechanism, in stark contrast to the conventional engineering materials, where the principal focus has been on the simple enhancements in the strength and stiffness. In fact, the value of these features has been overlooked in commercial composite filaments for fused deposition modeling (FDM). The effective fiber length and the affinity of the fibers to the matrix are important engineering factors in the manufacture of short fiber composites, and the effect can be further extended in the sequential and hierarchical 3D printing process.

**Figure 2:**
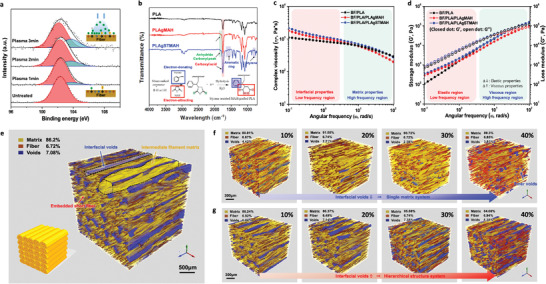
Material designs for strong interfacial adhesion between the fiber and the matrix. a) Formation of O_2_/Ar plasma induced‐polar groups at the fiber surface. The inset describes the change in chemical compositions on the fiber surface before and after plasma treatment. b) Formation of hydrophilic grafting functional molecules in the polymer matrix. The inset shows the mechanism of ST‐assisted grafting polymerization of MAH to PLA. (See Figure S1, Supporting Information, for detailed mechanism.) c) Complex viscosity (η*, Pa*s) of the molten composites with respect to the frequency. d) Storage modulus (*G′*) and loss modulus (*G″*) curves with respect to the frequency. e) Representative 3D tomographic images of hierarchical SFRP composites inherently induced by the 3D printing process. f, g) Resultant tomographic images comparing the effects of rheological modification on the internal microstructure of the composites according to the contents of 10, 20, 30, and 40 wt% of f) PLA‐g‐MAH and g) PLA‐g‐STMAH. (The formation of a high number of isolated inner voids is attributed to poor processing conditions and is accompanied by a significant change in the viscosity.)

As described in **Figure** **2**, our study starts from the effective interfacial adhesion between the fiber and matrix. Basalt fiber (BF), eco‐friendly natural fiber with a chemical composition and structure similar to those of glass fiber, was used as reinforcement. An O_2_/Ar plasma treatment was applied to clean the BF surface and activate dangling surface bonds for modification of the chemical composition of the surface. Plasma was ignited with Ar gas in a vacuum chamber, and O_2_ gas was supplied to radicals on the activated surface to form various types of active oxygen species. These newly formed oxygen species could induce to form strong intermolecular hydrogen bonding with the matrix resin. The X‐ray photoelectron spectroscopy (XPS) narrow scan for Si2p clearly shows the induced oxygen functionalities (Figure 2a). The newly emerging peak at 103.88 eV is attributed to the chain structure of [Si_2_O_6_]^4−^ at the surface, while the pre‐existing peak at 101.62 eV corresponds to the tetrahedral structure of [SiO_4_]^4−^ in the bulk phase. The appearance of the new peak in the high binding energy area well supports the formation of the oxygen species on the BF surface.

Subsequently, strong interfacial bonding can also be enhanced by introducing hydrophilic moieties in the PLA matrix resin that are capable of chemical coupling with the oxygen species at the BF surface. Grafting of high reactive functional moieties onto the PLA chain can be suggested as an effective way on this purpose.^[^ ^49^ ^]^ Maleic anhydride (MAH) monomers with polar functional groups were grafted onto macro‐radical sites along the backbone of PLA by means of radical initiators. Styrene monomers (here, ST in the notation) with an electron‐donating character were utilized to promote the interaction with MAH monomers (having an electron‐attracting character), via charge transfer complex formation and subsequent copolymerization. Along with the assistance from ST co‐monomers, these MAH monomers easily reacted with the macro‐radicals, which not only improved the grafting density of MAH monomers but also diminished the degradation of the PLA matrix (see Figure S1, Supporting Information).

Figure 2b shows Fourier transform infrared (FTIR) spectra of pure PLA, PLA grafted with MAH (PLA‐g‐MAH), and PLA with ST‐assisted grafting of MAH (PLA‐g‐STMAH) over the wavelength of 600–4000 cm^−1^. A strong intensity peak for the carboxylic acid group was detected at 1720 cm^−1^, in addition to a shoulder peak assigned to the carbonyl group (C═O) of MAH at 1780 cm^−1^. The extra peak (at 850 cm^−1^) attributed to the phenyl group of ST indicates the ST‐assisted MAH grafting polymerization. The wavelength of 1500–1600 cm^−1^ also shows newly emerging peaks representing the aromatic ring of ST. The ^1^H NMR spectrum of the molecular structure of the ST‐assisted MAH‐grafted polymer, verifying the FTIR results, was presented as well (see Figure S2, Supporting Information). The spectrum showed a weak peak intensity due to aromatic protons in the range of 6.9–7.4 ppm and characteristic peaks due to the succinyl functional groups of MAH in the range of 2–3 ppm, respectively.^[^ ^50^, ^51^ ^]^ DSC thermal study also showed that the thermal parameters such as crystallization and melting temperature of PLA decrease after MAH and St/MAH grafting, indicating that the crystallization of PLA was limited to steric hindrance of the grafted pendant^[^ ^52^, ^53^ ^]^ (Figure S3, Supporting Information). Figure S4, Supporting Information, shows that the compositional ratio of ST to MAH strongly influenced the grafting efficiency and the melt flow index (MFI) of PLA upon the MAH grafting process. When the MAH was solely engaged in the grafting polymerization (i.e., without ST), the chain degradation of PLA was severe, thus greatly reducing the melt viscosity. By contrast, ST‐assisted MAH grafting stabilized the PLA backbone chain and relieved the chain scission. This indicates that the ST monomers serve as a rheology modifier for the 3D printable structure. GPC results showed a dramatic change in the molecular weight of PLA due to hydrolysis during graft polymerization (see Figure S5, Supporting Information). The results suggested good control of the ST‐assisted MAH graft polymerization showing a relatively minor decrease in molecular weight with a narrow dispersion (PDI = 2.17).

A rheological study was carried out for the composite precursor system prepared by blending PLA‐g‐MAH and PLA‐g‐STMAH with BF/PLA as a function of mass fraction ratio. Figure 2c shows the shear rheological responses of the composites in their molten state, monitored to examine the effect of interfacial enhancement on the viscoelastic properties; various properties of the molten composites are presented to show the level of viscosity, shear‐thinning behavior, and the interfacial properties between the fibers and the matrix. For the BF/PLA/PLA‐g‐STMAH system, complex viscosity (ƞ*) tends to increase, especially in the low‐frequency region, signifying the presence of a strong interaction between BF and PLA. Obviously, this response is consistent with the strong interfacial hydrogen bonding between the plasma induced‐polar groups of BF and the grafted reactive functional groups of PLA matrix. In the high‐frequency region, a strong shear thinning was evident while the chain entanglement of the grafted branches gradually relaxed, allowing for the orientation in the flow direction.

Figure 2d shows the dynamic viscoelastic behaviors represented by the storage modulus (*G′*) and loss modulus (*G″*) with respect to the measuring frequency. With the addition of PLA‐g‐STMAH, the *G′* and *G″* axes slightly shifted upward and the slopes of *G′* and *G″* with respect to the frequency decreased, showing diminished viscous behavior and enhanced elasticity. The high elastic response over the entire frequency region is attributed to the increase in grafted branches. Meanwhile, the BF/PLA/PLA‐g‐MAH system showed that the *ƞ*, *G′*, and *G″* increased in the low‐frequency region but decreased in the high‐frequency region. The decrease in the high‐frequency region was due to the significant reduction in the molecular weight of PLA upon the grafting polymerization that imparts a high melt fluidity but a loss in elastic fluid‐like behavior, thereby making it difficult to form a stable 3D architecture in the molten state.^[^ ^54^, ^55^ ^]^

The variation of viscoelastic behavior can lead to the development of different internal microstructures in the composites. Figure 2e–g shows 3D microtomographic images of the composites obtained by X‐ray micro‐computed tomography measurements. Cross‐sectional tomographic images of the composites represent their additive building architecture, in which 0.3‐mm‐thick layers were successively printed in the longitudinal direction, that is, from the bottom to the top layers following the tensile direction; Each building element, including the fiber, void, and matrix phases, was colored differently (Figure S6, Supporting Information). Upon a 3D printing process, the material under deposition must pass through a narrow nozzle in the molten state and, thus, experience a high shear force exerted at nozzle walls. This induces an anisotropic microstructure composed of filament threads, embedded fibers, and void channels, all highly aligned in the printing direction (Figure 2e). The spherical coordinate mapping images of orientation distribution of fiber, voids, and matrix show a high concentration in one spot corresponding to a specific direction^[^ ^33^ ^]^ (Figure S7, Supporting Information).

For BF/PLA/PLA‐g‐STMAH system, the resultant structure formed a series of fibrous assemblies hierarchically built with abundant intermediate filament bundles (Figure 2g). 3D printing yielded a series of parallel filament threads stacked layer‐by‐layer, among which sequential air gaps are formed in the shape of a triangular prism throughout the entire volume. Each filament thread contained highly ordered embedded fibers. These resulted in an overall anisotropic geometry induced by the processing direction.

Noteworthy that BF/PLA/PLA‐g‐MAH system does not show interfacial void channels among inter‐filament threads; thus, the microstructure formed a single matrix composite system (Figure 2f). In terms of negative void elimination, it is a favorable result. This is due to the intrinsic low viscosity of depositing precursor that freely flows filled the voids among the inter‐filaments. Unfortunately, this easy flow of melt is related to the significant decrease in the molecular weight upon the graft polymerization. Excessive molecular weight reduction can contribute to a poor shape‐forming capability and eventually result in weak structural strength and brittle nature.

**Figure 3:**
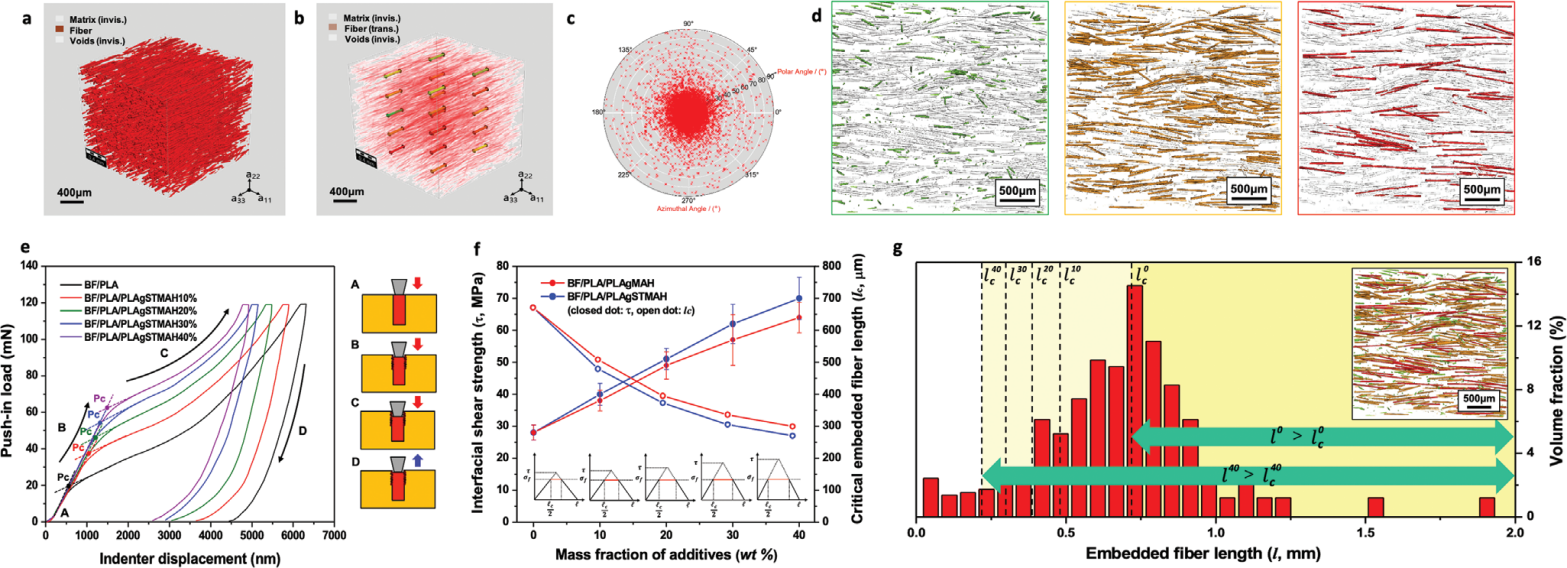
Effective fiber architecture designs for simultaneous improvements of strength, stiffness, toughness in composites. a) Segmentation images of individual fibers to quantify the fiber length, position, and orientation. b) Localized fiber orientation with representative directional arrows in each region block. c) Fiber orientation mapping image in the spherical coordinate system. d) Color‐coded tomography images to verify the fiber length distribution with respect to *l*_c_; red‐color fibers (*l* > $l_{c}^{40}$), yellow‐color fibers ($l_{c\;}^{0}$> *l* > $l_{c}^{40}$), and green‐color fibers (*l* < $l_{c}^{0}$). e) IFSS (*τ*) between fiber and matrix was evaluated with push‐in tests using nanoindentation. Load‐displacement curves compose of four segments: the initial load segment (A) that represents incomplete contact between the indenter tip and the fibers, the linear region (B) where the fibers and matrix deform elastically under a load, the nonlinear region (C) where the slope starts to decrease following the onset of interfacial failure under load, and (D) the unloading curve. f) Correlation between *l*_c_ and *τ*. Stress‐position profiles show correlation between *l*_c_ and *τ*. g) Representative fiber length distribution (*l*). $l_{c}^{10}$–$l_{c}^{40}$ denote each boundary line axis of *l*_c_ in composites with PLA‐g‐STMAH of 10–40 wt%.

**Figure** **3**a–d shows the segmentation images of individual fibers to quantify their shape, length, position, and orientation. The fiber segmentation image shows the unidirectional alignment of fibers distributed throughout the matrix, resulting in an anisotropic architecture induced by the printing direction (Figure 3a). The main fiber orientation tensor in the entire system: *a*_11_ = 0.0363, *a*_22_ = 0.0542, *a*_33_ = 0.9094, indicates a directional vector along the *Z*‐axis as high as 0.9 (Figure 3b). The spherical coordinate system presents 2D mapping images of the fiber orientation distributions. These results revealed that a substantial amount of fiber was positioned in the *XZ*‐plane and was highly oriented along the *Z*‐axis direction, which corresponds to the printing direction (Figure 3c). The shear‐induced fiber architecture exhibits anisotropy along the printing direction, in contrast to the isotropic fiber architecture in composite materials prepared by conventional molding processes.^[^ ^33^ ^]^ In the meantime, many methodologies have been used, including 3D printing and other fiber infiltration, to realize well‐ordered fiber‐reinforced composites. Recently, it is reported that 3D printing fabricates a sequential additive architecture of intermediate materials with highly aligned fiber inside induced by shear force and electromagnetic fields. Due to the merit in design freedom, mass customization, and fabrication feasibility, 3D printing has a special advantage of tailoring the fibers to a preferred orientation within a complex structure compared to other methods.^[^ ^56^, ^57^, ^58^, ^59^ ^]^

The effectiveness of interfacial treatment was evaluated with push‐in tests using nanoindentation.^[^ ^60^, ^61^ ^]^ Figure 3e shows the resultant load–displacement curves obtained while an axial load was applied until the interfacial failure between fiber and matrix. The IFSS was determined based on the critical load (*Pc*) at the onset of interfacial failure. (Figure S8, Supporting Information, details the schematic illustration and indentation curves of interfacial crack propagation in nanoindentation push‐in tests.) Following the interfacial failure, the fibers were pushed into the matrix to a certain depth, that is, load depth, as shown in the schematic images of A–D in Figure 3e. Interestingly, remarkable interfacial improvement was achieved, with a dramatic increase in the IFSS between the fiber and matrix over 250%. This interfacial improvement was attributed to intermolecular hydrogen bonding between polar functional groups in fiber and matrix. In the BF/PLA specimen, an inflection point, which indicates the onset of interfacial failure, was observed at *Pc* = 20 mN; by contrast, in the BF/PLA/PLA‐g‐STMAH 40 wt% specimen, the onset of interfacial failure reached up to *Pc* = 62 mN, corresponding to an IFSS value of 70 MPa (Figure 3f).

IFSS value determined here was used to define its relationship with respect to the critical length (*l*_c_) of the embedded fiber. For the fiber length over *l*_c_, the strength and modulus of the fiber can be fully transferred to the matrix resin, such that composites can be effectively reinforced. IFSS is inversely proportional to *l*_c_; thus, as the interfacial adhesion improves, the *l*_c_ of the embedded fiber becomes shorter. Figure 3d,g shows the fiber length distribution, where BF with an initial length of 3 mm had undergone significant breakage throughout the entire 3D printing process; thus, the final embedment length of BF was ≈700 µm. When *l*_c_ was substituted into the fiber length distribution, the boundary line axis, which represents *l*_c_, tended to shift to a lower value according to the interfacial improvement. (Here, we assume that all samples have the same fiber length.) This means that the higher the IFSS, the greater the ratio of fibers over *l*_c_. The BF/PLA/PLA‐g‐STMAH 40 wt% specimen had a much higher IFSS value, as well as larger amounts of fibers with the embedment fiber length longer than *l*_c_ (corresponding to the right region of $l_{c}^{40}$), despite the same length with the BF/PLA specimen. (The fiber length distribution of BF/PLA with *l* > *l*_c_ corresponds to the right region of $l_{c}^{0}$). In the next session, we will discuss in detail the mechanical behavior of composites with effective fiber length based on the correlation between IFSS and *l*_c_.

**Figure 4:**
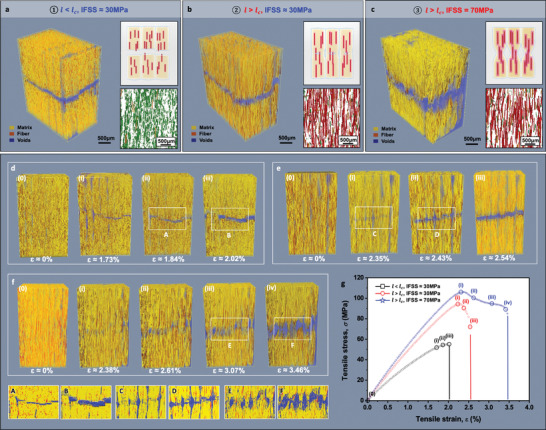
In situ tomographic observation on 3D‐printed SFRP composites under tensile loading condition. a–c) Representative 3D tomographic images showing final failures of 3D‐printed hierarchical BF/PLA/PLA‐g‐STMAH composites. Various deformation and fracture modes based on fiber length and interfacial conditions; a) ① *l* < *l*_c_, *l* ≈ 50 µm, IFSS ≈ 30 MPa; b) ② *l* > *l*_c_, *l* ≈ 700 µm, IFSS ≈ 30 MPa; and c) ③ *l* > *l*_c_, *l* ≈ 700 µm, IFSS = 70 MPa. Color‐coded tomography images showing fiber length distribution inside. Given as red‐color fibers (*l* > 600 µm), yellow‐color fibers (300 µm > *l* > 600 µm), and green‐color fibers (*l* < 300 µm). d–f) Detailed illustration of the progressive failure process at each tensile loading sequence for 3D‐printed hierarchical BF/PLA/PLA‐g‐STMAH composites. Progression of the deformation and fracture modes of specimens based on fiber length and interfacial conditions; d) ① *l* < *l*_c_, *l* ≈ 50 µm, IFSS ≈ 30 MPa; e) ② *l* > *l*_c_, *l* ≈ 700 µm, IFSS ≈ 30 MPa; and f) ③ *l* > *l*_c_, *l* ≈ 700 µm, IFSS = 70 MPa. 2D extended images A–F correspond to the marked regions for A–F in (d–f), respectively. g) Representative stress–strain curves of 3D‐printed hierarchical SFRP composites showing each tensile loading sequence at which a fractographic image was acquired with X‐ray microscopy. Circle points represent the strains corresponding to the fractographic images in (d–f), respectively.

In situ X‐ray tomography enabled the detailed examination of the tensile fracture behaviors to resolve the concurrent reinforcement mechanism for strength, stiffness, and toughness. **Figure** **4** and Figure S9, Supporting Information, compare the different tendencies of deformation and fracture behaviors of BF/PLA/PLA‐g‐MAH and BF/PLA/PLA‐g‐STMAH. The two different systems of single matrix composites versus hierarchical composites lead to the distinctively different fracture mechanisms, from fiber pull‐out to fiber bridging, and ultimately to matrix deformation. Figure 4a–c (or Figure S9a–c, Supporting Information) shows representative fractographic images of 3D‐printed SFRP composites upon the final failure at the tensile limit. Figure 4a,b (or Figure S9a,b, Supporting Information) shows the influences from critical embedment fiber length (*l*_c_), which enables an effective strength enhancement of the composites, on fracture behavior. Color‐coded tomography images in Figure 4a–c (or Figure S9a–c, Supporting Information) provide fiber length distribution inside. In terms of interfacial analysis, we also investigated the micro‐scale fracture behavior locally occurring at the interface between the fiber and the matrix with respect to their interfacial adhesion level, as shown in Figure 4b,c (or Figure S9b,c, Supporting Information).

We first attempted to understand the tensile fracture behavior in BF/PLA/PLA‐g‐MAH system, which has a single matrix microstructure similar to the typical composites, as shown in Figure S9, Supporting Information. When the fiber length was shorter than *l*_c_, the local shear stress between the fiber and the matrix failed to reach the critical value, such that the fiber was easily pulled out from the matrix (see Figure S9d, Supporting Information). (The results were the same even when the interfacial adhesion was improved.) By contrast, when the fiber length was over *l*_c_, the localized deformation induced by the embedded fibers was clearly observed in the matrix (Figure S9e, Supporting Information). Due to weak interfacial adhesion, an initial crack started to propagate from the weak interface between the fibers and matrix, and critical voids became appeared near the interface. As the tensile load increased gradually, the weak fiber–matrix adhesion led to the interfacial failure, where the matrix was de‐bonded from the fiber surface (see Extended images A–C in Figure S9e, Supporting Information). When a strong interface is formed, a significantly different fracture behavior is observed, as shown in Figure S9f, Supporting Information. During the first load sequence, sharp cracks occurred not at the fiber–matrix interface but inside the matrix. Subsequently, cracks started to propagate in a straight mode perpendicular to the applied load; however, interfacial failure was not observed. During the third load sequence, fiber bridging, which is indicative of strong fiber–matrix adhesion, was commonly observed. In the last load sequence, where the final failure occurred, a significant amount of bridged fiber reached fracture (see Extended images D–F in Figure S9f, Supporting Information). These results can be better understood by a reinforcement mechanism based on the relationship between *l*_c_ and the fracture mode, in which *l* < *l*_c_ corresponds to the fiber pull‐out mechanism and *l* > *l*_c_ describes the fiber bridging mechanism. According to this reinforcement mechanism, high stiffness can be achieved, yet accompanied by a brittle weakness (Figure S9g, Supporting Information). The results above are considered highly satisfactory from the existing perspectives on composites but, due to its brittle nature, there are inherent limitations for the application in practical engineering fields that require simultaneous strength, stiffness, and toughness.

Figure 4 presents the distinctive tensile failure behaviors for BF/PLA/PLA‐g‐STMAH system. Remarkably, this composite system exhibited a microstructure‐dependent behavior significantly different from the typical counterparts. When the embedded fiber length was shorter than *l*_c_, it exhibited a simple transverse fracture (Figure 4d). By contrast, for the fiber length over *l*_c_, severe localized matrix deformation was observed, originating from the shear stress of the surrounding embedded fibers (Figure 4e). The geometric nature of interfacial void channels partitioning the boundary between each filament thread acted as a specific condition for localized matrix deformation at the surrounding areas of fibers under the local shear stress. Notably, when fiber–matrix interface is sufficiently strong, shear yielding occurred in the matrix, causing the matrix polymer to be highly stretched in the tensile load direction (Figure 4f). That is, necking eventually occurred. The necking behavior appeared in individual filament threads, as the greatest local stress was concentrated in this region, and eventually evolved into fracture when a critical strain was reached (see Extended images E and F in Figure 4f). In this regard, we can find a similar fracture behavior in the toughness mechanism of bamboo fiber with reference to the hierarchical structure and mechanical properties of individual bamboo components. Recent studies reported that fiber bundles in bamboo achieve high strength and toughness with crack bridging and fiber pull‐out against applied loads.^[^ ^29^, ^30^ ^]^

**Figure 5:**
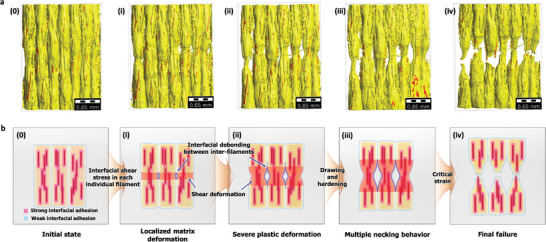
Detailed illustration of representative failure mechanism of 3D‐printed hierarchical BF/PLA/PLA‐g‐STMAH composites. a) 3D tomographic images showing progressive failure process at each tensile loading sequence; (0) initial state, (i) first load sequence, (ii) second load sequence, (iii) third load sequence, and (iv) final failure. b) Comprehensive description of deformation and fracture progression.

The interesting failure mechanism of BF/PLA/PLA‐g‐STMAH can be understood in detail by the following mechanism (see **Figure** **5**). Given that individual filament threads were partially partitioned by the interfacial void channels, each filament thread was fully subjected to the local range of shear stress caused by the embedded fibers, highly oriented in the tensile load direction. Besides, the effective fiber length, where the fiber length exceeds its critical value to achieve effective interfacial bonding, leads to a high level of shear stress at the interface between the fibers and matrix (see loading sequence (0)–(i)). During this progress, the strong interfacial adhesion between fiber and matrix resulted in the localized matrix deformation in the surrounding areas of fibers (see loading sequence (i)–(ii)). Simultaneously, the weak inter‐filament interfacial adhesion allowed such localization to be a severe plastic deformation throughout the matrix. This mechanism eventually evolved into necking behaviors (see loading sequence (ii)–(iii)), in each filament thread. Consequently, multiple localized necking behavior occurred in most of the intermediate filament threads and resulted in the enhanced fracture toughness while the material had reached the yield strength and underwent drawing and hardening in plastic deformation (see loading sequence (iii)–(iv)). In terms of the interfacial failure, this behavior is closely related to the heterogeneous interfacial adhesion system, consisting of the strong interfacial adhesion between the fiber and the matrix along with the weak interfacial adhesion among intermediate filament threads. This heterogeneous interface structure mimics the localized heterogeneity of natural architectures and has a great potential to initiate toughness reinforcement mechanisms.

**Figure 6:**
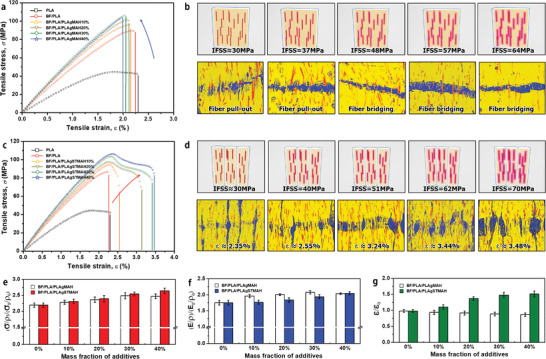
Comparative results of tensile strength and its stress–strain curve of 3D‐printed composites according to the increment of interfacial strength. a) Representative stress–strain curve of 3D‐printed single matrix BF/PLA/PLA‐g‐MAH composites showing typical mechanical behavior of engineering materials that exhibit stiff but brittle nature. b) Resultant failure mechanism transition from fiber pull‐out to fiber bridging to achieve stiffness reinforcement as interfacial strength increases. c) Representative stress–strain curve of 3D‐printed hierarchical BF/PLA/PLA‐g‐STMAH composites showing a simultaneous improvement in strength, stiffness, and toughness. d) Resultant failure mechanism showing multiple necking behavior to achieve toughness enhancement as interfacial strength increases. e–g) Comparative results of the specific tensile strength ((*σ*/*ρ*)/(*σ*_0_/*ρ*_0_)), specific tensile modulus ((*E*/*ρ*)/(*E*_0_/*ρ*_0_)), and maximum strain at break (*ɛ*/*ɛ*_0_) of the two composite systems.

**Figure** **6**a,c show the comparison of tensile strength and stress–strain curves of the 3D‐printed composites with an increment of interfacial strength between fiber and matrix. For the BF/PLA/PLA‐g‐MAH specimen, typical stress–strain behavior for conventional engineering composites is observed, such that its Young's modulus increased sharply, whereas the strain to failure decreased gradually, indicating a stiff but brittle system (Figure 6a). Interfacial improvement leads to a gradual transition of the failure mechanism from fiber pull‐out to fiber bridging (Figure 6b). By contrast, the BF/PLA/PLA‐g‐STMAH specimen showed distinct mechanical responses, where simultaneous improvements are highlighted in the strength, stiffness, and toughness (Figure 6c). The BF/PLA/PLA‐g‐STMAH system underwent significant localized deformation in the tensile direction, resulting in the necking effect. The phenomenon became more pronounced with interfacial improvement. Eventually, the brittle BF/PLA composites reached *ɛ* = 3.48% at IFSS = 70 MPa, with a noticeable increase in the toughness (Figure 6d). Figure 6e–g compare the results of the specific tensile strength ((*σ*/*ρ*)/(*σ*_0_/*ρ*_0_)), specific tensile modulus ((*E*/*ρ*)/(*E_0_*/*ρ*_0_)), and maximum strain at break (*ɛ*/*ɛ_0_*) between the two systems. The data was normalized by the respective values of the base material. The results showed that the specific tensile strength and modulus tend to increase steadily as a function of the interfacial bonding strength between fiber and matrix. As the interfacial strength increased, the BF/PLA/PLA‐g‐STMAH system, which initially showed a low tensile strength, exceeded the BF/PLA/PLA‐g‐MAH system, eventually reaching a more than twofold increase in specific tensile strength compared to the base material. In terms of specific modulus, the two systems came to a similar level of modulus value, despite differences in internal void ratio. This means that the interfacial voids work as a function of structural elements rather than negative defects, in the BF/PLA/PLA‐g‐STMAH system. A new point of our finding here is that these structural voids lead to a concomitant increase in elongation rate. The BF/PLA/PLA‐g‐STMAH system clearly exhibited gradual increases in tensile strength and modulus with an exceptional increase in maximum strain at break (up to 1.5 times).

## 3 Conclusion

In summary, we have demonstrated the distinctive mechanical behavior of hierarchical composite systems, originating from the intrinsic feature of the 3D printing process, which can be characterized not only by the structural anisotropy and hierarchical multilevel structure but also by the heterogeneous interfacial adhesion system among the constituting elements. Indeed, the BF/PLA/PLA‐g‐STMAH composite system showed a noticeable increase in the tensile strength and Young's modulus by more than twofold, together with further elongation. Our report also added that it shows remarkable strength and toughness increments even at the low short fiber content of 5–10 vol%. This slightly differs from the conventional engineering perspective, where current studies aim to achieve high fiber content impregnation with continuous fibers. Such research has shown that 3D printing of continuous fiber‐reinforced composites cannot promise preferable mechanical performance due to the inevitable void formation and weakened interlayer bonding compared to conventional engineering composites.^[^ ^37^, ^38^, ^39^ ^]^ It is also hard to expect the strain hardening plastic deformation and ensuing toughness improvement in continuous fiber composites, given composite's high dependence on the physical properties of fibers.^[^ ^28^, ^40^ ^]^ Short fiber‐reinforced polymer composites, by contrast, have shown a synergistic integration between the strength of the fiber and the toughness of the polymer matrix. Noteworthy that the composite system we proposed here shows a high specific strength in the presence of voids among filament assemblies. Moreover, strategic incorporation of the interfacial voids enables toughness reinforcement mechanism, which can be understood from nature‐inspired biological systems. To the best of our knowledge, this is the first insight into 3D‐printed short fiber‐reinforced composites with an expanded understanding of nature‐inspired architecture. This new perspective will help to address relevant issues facing this technology in a more flexible manner and to broaden the scope of 3D printing into a more extensive range of research and engineering applications.

## 4 Experimental Section

### Materials

BF in a form of the chopped strand with an average diameter of 12 µm and an average length of 3 mm was used as reinforcements and provided by Kamenny Vek, Moscow, Russia. BF is a fine fibrous material made from natural volcanic rock and is an environmentally friendly and cost‐effective alternative to glass fiber. BF has similar physicochemical properties to glass fiber but shows better thermal and mechanical properties than glass fiber. Poly lactic acid (PLA) in pellet form, an eco‐friendly feedstock material for the FDM‐type 3D printing process, was used as the matrix resin and purchased from Nature Work Co., China. PLA is a semi‐crystalline thermoplastic polymer with 15 g/10 min (2.16 kg, 190 °C) of the MFI and 1.24 g cm^−3^ of the density. MAH (98%) and ST monomer as grafting functional monomers were supplied by Daejung Chemical Co., Korea. Dicumyl peroxide (97%) as a free radical initiator was purchased from Aldrich Co., USA. Dimethylformamide (DMF, 99.9%) as an organic solvent and acetone (99.5%) as a washer for the grafted product were purchased from Daejung Chemical Co., Korea.

### O_2_/Ar Plasma Treatment

For the surface treatment of BF, plasma was discharged using a high‐frequency RF generator of 13.56 MHz. BF was prepared in a vacuum chamber, the chamber was degassed down to 30 mTorr, and then Ar gas was injected to induce the plasma, and the fiber surface was modified with O_2_ gas. Ar gas and O_2_ gas flowed into the chamber at 100 sccm, and were maintained for 3 min. The plasma power was set at 200 W and a matching network was connected to match the voltage and current phases to supply stable power.

### Grafting Polymerization of ST/MAH Monomers

Grafting reactions were carried out in a flask equipped with an impeller, thermocouple, and reflux condenser. PLA was dissolved in DMF at 140 °C. The mixture containing a certain amount of PLA in the solvent was heated in a heating mantle to dissolve the polymer. When the dissolution was completed, a certain amount of MAH, ST monomer, and initiator were dissolved in 100 mL solvent and added to the mixture. The time of addition of MAH, ST, and initiator was recorded as the starting time of the reaction. After reaching the desired time, the reaction was stopped and the samples were cooled down at room temperature until the completion of recrystallization. It was then precipitated in 300 mL of acetone, repeatedly washed with acetone to remove unreacted monomers and homopolymers. Finally, it was filtered and dried in a vacuum oven at 80 °C to a constant weight.

### Fabrication of 3D Printable BF/PLA Composite Filaments

3D printable BF/PLA composite filaments were fabricated by a co‐rotating, intermeshing twin‐screw extruder with an inner diameter of 24 mm and L/D of 2 mm (Haake extruder; Thermo Fisher Scientific, Waltham, USA). During the extrusion, the barrel temperature was 220 °C, and the screw rotational speed was set at 60 rpm. Special screw configuration was designed to improve efficiency in the manufacturing of short fiber‐reinforced composites. It consists of two mixing zones: a dispersive mixing zone and a distributive mixing zone. During the mixing in a molten state, neat PLA resin was fed through the hopper and transferred into the dispersive mixing zone, and the grafted molecules (PLA‐g‐MAH and PLA‐g‐STMAH) were fed through the 1st side feeder into the mixing zone at a ratio of 10, 20, 30, and 40 wt%, respectively. Subsequently, in the distributive mixing zone, the fibers were fed at a ratio of 6–7 vol% through the 2nd side feeder to uniformly distribute the fibers throughout the matrix resin without severe fiber damage. The mixed composites were extruded into a continuous filament thread from a cylindrical die with a 1.75 mm diameter, and quickly solidified by dry air and wound onto a spool using a winding machine. The prepared filaments were used as feedstock materials for the FDM‐type 3D printing process.

### Printing of BF/PLA Composites

The 3D printing procedure was carried out according to the following three steps: specimen modeling, slicing, and printing. In accordance with ASTM D638 Type V, dog bone‐shaped tensile specimens were designed with a computer‐aided modeling package (CADian3D, IntelliKorea Co., Korea). The detailed geometric specifications were as follows. The overall length (LO) and width (WO) of the specimen were 24.6 and 6.8 mm, respectively, and the length (L) and width (W) of the narrow part of the specimen were 4.2 and 3.4 mm, respectively, and the thickness was 1.8 mm. Once the specimen was created as designed, the slicing software (Simplify3D, USA) converted the digital 3D model into a series of thin layers and produced printing instructions tailored to a specific type of printer. The thickness of each layer was set to 0.3 mm, and the infill density, which means the amount of filament printed inside the object, was set to 100%. After that, each specimen was fabricated by the FDM‐type 3D printer (3DISON AEP, Rokit Co., Korea). To build up 3D architecture, the printer nozzle was positioned as programmed, while being heated to 220 °C and the bed temperature was kept at 60 °C during operation. Subsequently, the nozzle deposited molten filament threads, and these thin threads were printed on top of each other at a 50 mm s^−1^ printing speed until the desired shape was achieved. The printing path with a 90° raster angle was set parallel to the tensile loading direction. As such, a total of six layers were printed layer by layer in that direction.

### Quantitative Characterization of the Treated BF

The change in the chemical composition of the BF surface was analyzed by XPS using a Thermo Fisher Scientific ESCALAB 250Xi XPS instrument with monochromatic Al‐K*α* X‐radiation. The functional groups formed to the BF surface were determined by FTIR using an FT/IR‐4600 instrument (JASCO, Oklahoma City, OK, USA). The surface roughness and topography of the BF surface were analyzed by atomic force microscopy (AFM) using an NX10 AFM instrument (Park Systems, Suwon, South Korea).

### Grafting Degree Determination

The MAH content of the grafted PLA and reaction products was assessed using colorimetric titrations of xylene and refluxed under stirring and controlled temperature until a clear solution was obtained. To hydrolyze the anhydride function, the solution temperature was lowered below 100 °C and then, a certain amount of distilled water was added to the solution, and refluxing was continued for a period of time to ensure that the carbonyl groups were converted to acid groups. The solution of 0.1 N ethanolic KOH was used to determine the acidity of the grafted polymer with a thymol blue indicator until a deep blue color was obtained. The acid number and grafting degree (GD) was calculated by the following equation.

(2) $$\mathrm{Acid}\;\mathrm{number}\;=\;\frac{V_{\mathrm{KOH}}\;\times \;N_{\mathrm{KOH}}\;\times \;56.1}{W_{\mathrm{gPLA}}}$$

(3) $$\mathrm{GD}\;\;\left(\%\right)=\;\frac{W_{\mathrm{gMAH}}}{W_{\mathrm{PLA}}}\;\times \;100\;=\;\frac{\mathrm{Acid}\;\mathrm{number}\;\times \;98}{2\;\times \;561}$$

where *V*_KOH_ and *N*_KOH_ are the consumed volume (mL) and equivalent concentration (mol L^−1^) of KOH solution in ethanol, respectively. *W*_PLA_ is the weight of PLA before grafting MAH and *W*_gPLA_ is the weight of PLA grafted with MAH after grafting MAH. *W*_gMAH_ is the weight of MAH grafting on PLA.

The percentage conversion also was calculated as follows.

(4) $$\mathrm{Conversion}\;\left(\%\right)=\;\frac{W_{\mathrm{gMAH}}}{W_{\mathrm{MAH}}}$$

where *W*_MAH_ is the weight of MAH initially taken on graft polymerization.

### Rheological Behaviors of the Composites

In accordance with ASTM D1238, the MFI of the composites was measured under a 2.16 kg load in a MFI apparatus from Davenport, UK, at a temperature of 190 °C. The measurements were performed in triplicate. The rheological investigations of the composites were conducted with the use of a rotational melt rheometer with a 25 mm diameter parallel plate, Discovery HR‐2, TA Instruments, New Castle, USA. The measurements were carried out in frequency sweep mode by varying the angular frequency from 10^−1^ to 10^2^ rad s^−1^ at an isothermal temperature of 190 °C. Frequency sweep experiments were performed at a strain value of 1%, in the linear viscoelastic region.

### Nano‐Indentation Push‐In Test

The push‐in test was conducted using a nano‐indentation instrument (Anton Paar, Nano‐indentation Tester NHT^3^, Austria) with a sharp tip of 5 mm in diameter. For the push‐in test, the specimens were cut to the surface perpendiculars to the fibers and mounted in epoxy resin to facilitate sample handling during polishing. The rough surfaces of the mounted samples were sequentially polished with 800, 1000, 1600, 2000, and 2400‐grit SiC papers, and finished with fine diamond pastes of 0.3 and 0.1 mm. After polishing the surface, the polished section was chemically etched to reveal the cross‐sectional area of embedded fibers for the micro‐mechanical test. Subsequently, each prepared sample was placed on metallic support to perform the fiber push‐in tests. The measurements were carried out for ten times per each sample under displacement load at 400 mN min^−1^.

The IFSS was determined based on the critical load value *P*c at the onset point where de‐bonding began to occur between the fibers and matrix resin during the push‐in tests.

(5) $$\mathrm{IFSS}\;=\;\frac{nP_{c}}{2\pi r^{2}}$$

where *P*c is the critical load, *r* is the fiber radius, and *n* is the parameter that depends on the elastic properties of the fibers and matrix. *n* can be obtained from the slope of the load‐displacement curve in the linear region, *S*_0_.

(6) $$n\;=\;\frac{S_{0}}{\pi rE_{f}}$$

where *E*_f_ is the longitudinal elastic modulus of the fiber.

### Tensile Test

The tensile strength, strain, and modulus of the 3D‐printed composite specimens were characterized by a universal tensile testing machine (Instron 5866, Norwood, MA) with a 30‐kN load cell. A tensile test was performed on dog‐bone‐shaped specimens with a crosshead speed of 0.5 mm min^−1^ at room temperature of 25 °C according to the ASTM D638 type V standard. The cross‐sectional area of the gage section was set to 9.0 mm^2^ and the gage length was set to 7.5 mm.

### X‐Ray µ‐CT Imaging

A high‐resolution X‐ray microscope (Xradia 520 Versa, Zeiss, Germany) was employed to acquire geometrical information about the internal microstructure of the BF/PLA composites. This X‐ray imaging system was equipped with a polychromatic tungsten X‐ray source and a high‐resolution detection system consisting of a scintillator, a light microscopy optic, and a charge‐coupled device, and used a two‐step magnification technique to provide submicron resolution even at large working distances. X‐ray source accelerated from the heated tungsten filament depending on the accelerating voltage. The scintillator absorbed X‐ray energy and converted it to visible light, which was then optically magnified. The detector collected emitted signals to form an image at different magnifications. The tomography workflow consisted of image acquisition and data processing steps. To acquire an X‐ray image, a sample was prepared and mounted according to the appropriate standard procedure. The sample holder assembly was mounted on the sample stage in equipment. The sample was aligned between the X‐ray source and detector, and then the X‐ray scan was initiated when reaching the appropriate position. 3D projection image was acquired by irradiating an X‐ray beam on the region of interest while rotating the sample by 360° from −180° to 180° at the set interval. During rotational scanning, both sample absorption and diffraction information were collected using the high‐resolution detection system. The operation of the imaging system was performed at an 4× low magnification under an acceleration voltage of 50 keV and power of 4 W. The region of interest was aligned to the center of the field of view using the stage positioning motors. The total projection image was 1601, and each projection image was acquired with an exposure time of 2.5 s and pixel binning of 2. The spatial resolution images with a voxel size of 4 µm were obtained by two‐stage magnification optics.

The data processing steps, which convert raw data into meaningful information through a process, were managed using a Scout‐and‐Scan control system. The collected projection images were separated from the background through segmentation and were then reconstructed into 3D geometric images using a Zeiss software package (Oberkochen, Germany). During the image reconstruction process, the motion drift of the specimen was corrected; various image artifacts were also corrected for precise analysis. After 3D reconstruction of the data, the results were visualized, refined, and finally exported for quantitative analysis. ORS Visual Pro (Object Research Systems, Canada) provided multi‐scale 3D imaging, high‐impact 3D visualization, and quantitative analysis for reconstruction image data. With this technical software tool, each building element of the composites, consisting of fibers, matrix, and voids, was segmented into different phases according to its gray level value determined by X‐ray transmittance.

### Tomography Image‐Based Simulation

3D tomography image‐based simulation was carried out using GeoDict, a commercial experimental software provided by Math2Market GmbH, Kaiserslautern, Germany. Among GeoDict's modules, FiberGuess and PoroDict were employed as the simulation tools for determining fiber identifications and voids observation, respectively. FiberGuess module identified short and long diameters of fibrous substances, and based on these results, determined the diameter and length of individual fibers. The orientation levels of embedded fibers were derived in the form of a second‐order fiber orientation tensor depending on the analysis of the in‐plane orientation angle (*ϕ*_f_) and out‐plane orientation angle (*θ*_f_). The existence of voids and cracks in the matrix was observed using the PoreDict module, analyzed with regard to the size, shape, and distribution, and schematically visualized.

### In Situ High‐Resolution X‐Ray Microscopy Tensile Test

In situ micro‐scale tensile testing was achieved to examine the deformation and failure mechanisms of 3D‐printed composites using high‐resolution X‐ray microscopy (Xradia 520 Versa, Zeiss, Germany) equipped with a micro‐test module. The micro‐test module consists of a tomography/load stage, a heating/cooling controller, and a load frame, containing a load cell, displacement motor, and upper and lower jaw assemblies. Especially, the Xradia Ultra Load Stage, capable of tension loading, offers new capabilities to observe internal failure processes including elastic and plastic deformation, crack initiation and propagation, and final failure down to the micron‐scale. The following describes the procedure for in situ determination. The sample was appropriately positioned on the stage by adjusting to the region of interest. Tomographic images were acquired while a specific level of the tensile load was applied to the sample. During the load, the stage maintained a constant temperature. This load sequence proceeded step by step until the specimen reached final failure. Tomography images were collected in all load sequences. The displacement speed of the tension crosshead was at 0.5 mm min^−1^, the same as the ASTM standards. To achieve scanning the fracture behavior of composites, the operation of X‐ray microscopy was conducted under an acceleration voltage of 50 keV and power of 3 W. During each scanning, the sample was rotated from −90° to 90°, and a total of 1601 projection images were collected with an exposure time of 2.5 s.

## Conflict of Interest

The authors declare no conflict of interest.

## Supporting information

Supporting Information

## Data Availability

Research data are not shared.
