# Supplementary material for: Outstanding Strengthening and Toughening Behavior of 3D‐Printed Fiber‐Reinforced Composites Designed by Biomimetic Interfacial Heterogeneity
Source: Adv Sci (Weinh). 2021 Nov 25;9(3):2103561. doi: 10.1002/advs.202103561 (PMC8787430; doi:10.1002/advs.202103561)
Supplement: Supplementary file 1 — Supporting Information [file ADVS-9-2103561-s001.pdf]

ADVANCED SCIENCE
Open Access

Supporting Information

for *Adv. Sci.*, DOI: 10.1002/advs.202103561

Outstanding strengthening and toughening behavior of 3D-printed fiber-reinforced composites designed by biomimetic interfacial heterogeneity

Siwon Yu, Yun Hyeong Hwang, Kang Taek Lee, Sang Ouk Kim, Jun Yeon Hwang*, Soon Hyung Hong**

Supporting information

Outstanding strengthening and toughening behavior of 3D-printed fiber-reinforced composites designed by biomimetic interfacial heterogeneity

Siwon Yu[a,b], Yun Hyeong Hwang[b], Kang Taek Lee[d], Sang Ouk Kim[c**], Jun Yeon Hwang[b**], Soon Hyung Hong[a,e*]

[a]Department of Material Science and Engineering, Korea Advanced Institute of Science and Technology (KAIST), 291 Daehak-ro, Yuseong-gu, Daejeon, 34141, Republic of Korea

[b]Institute of Advanced Composite Materials, Korea Institute of Science and Technology (KIST), Jeonbuk, 55324, Republic of Korea

[c]National Creative Research Initiative Center for Multi-dimensional Nanoscale Assembly, Korea Advanced Institute of Science and Technology (KAIST), 291 Daehak-ro, Yuseong-gu, Daejeon, 34141, Republic of Korea

[d]Department of Mechanical Engineering, Korea Advanced Institute of Science and Technology (KAIST), 291 Daehak-ro, Yuseong-gu, Daejeon, 34141, Republic of Korea

[e]Nanotechnology Research Institute, Jiaxing University, Jiaxing, China

*Corresponding author. Tel: (+82) 42 350 3327, E-mail: shhong@kaist.ac.kr

**Co-corresponding authors. Tel: (+82) 42 350 3339, E-mail: sangouk.kim@kaist.ac.kr, Tel: (+82) 63 219 8144, E-mail: Junyeon.Hwang@kist.re.kr

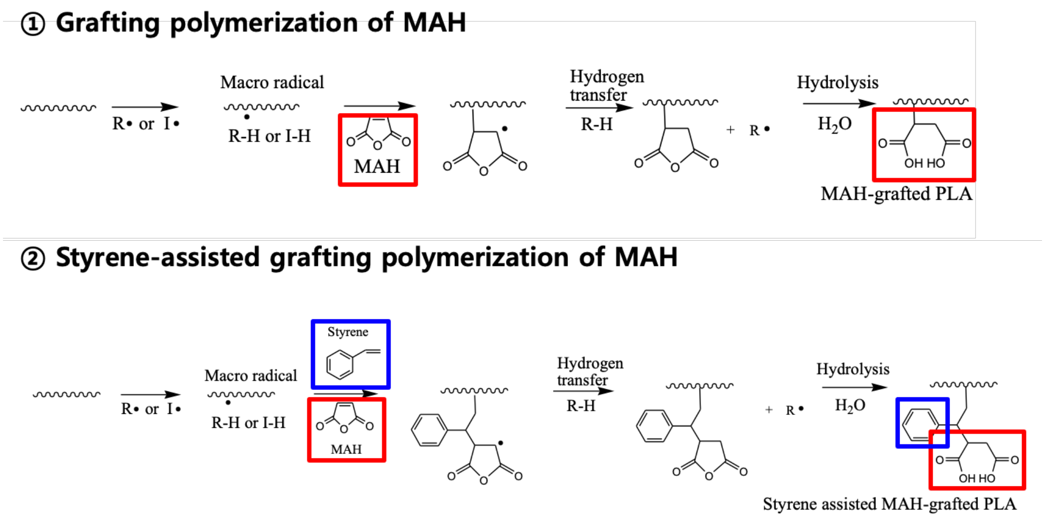

Figure S1. Mechanism of grafting polymerization of MAH onto PLA. ① grafting polymerization of MAH. ② styrene-assisted grafting polymerization of MAH.

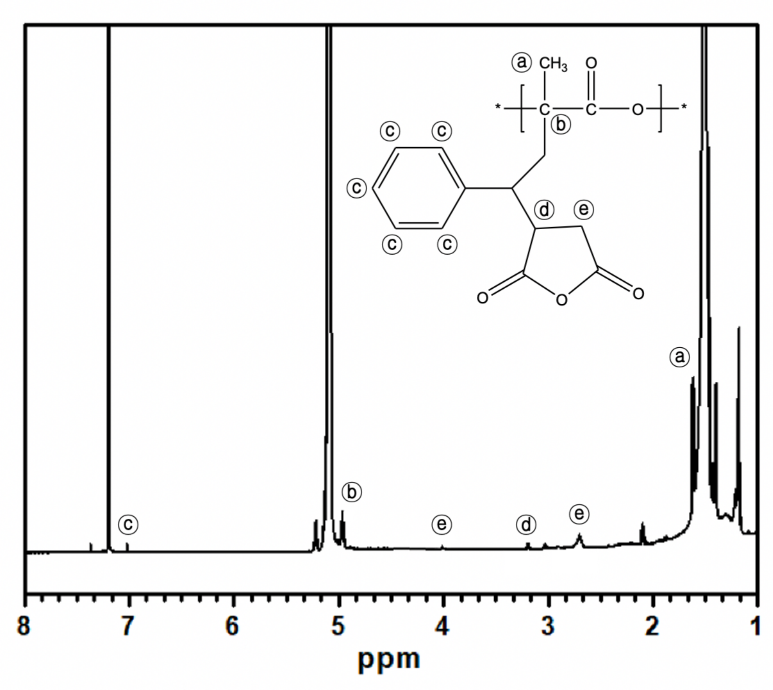

Figure S2. ^{1}H NMR spectra of the PLA-g-STMAH in $CDCl_3$. The assignment of protons is presented in the schematic of the molecular structure in the graph. The peaks of ⓐ (δ = 1.4-1.6 ppm) and ⓑ (δ = 5.1-5.4 ppm) are assigned to the proton of $–CH_3$ and -CH of the PLA backbone, respectively. The peak ⓒ (δ = 6.9-7.4 ppm) is assigned to the proton of styrene aromatic ring. The peaks of ⓓ and ⓔ (δ = 2-3 ppm) are assigned to the succinyl functional groups of MAH.

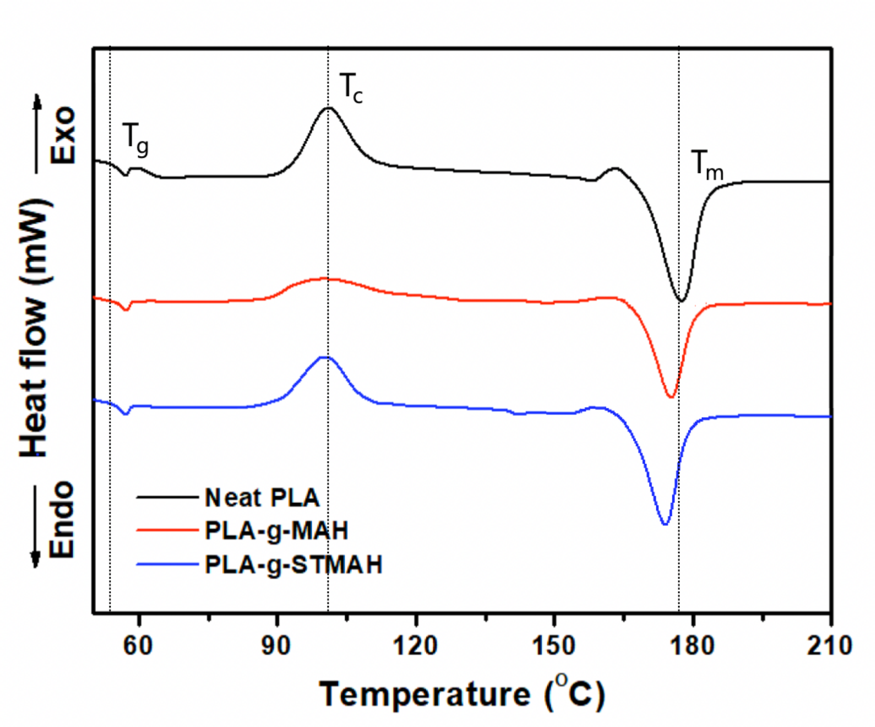

Figure S3. DSC thermograms of the neat PLA, PLA-g-MAH and PLA-g-STMAH showing the corresponding thermal parameters such as crystallization peak temperature (T_c), melting temperature (T_m) and glass transition temperature (T_g).

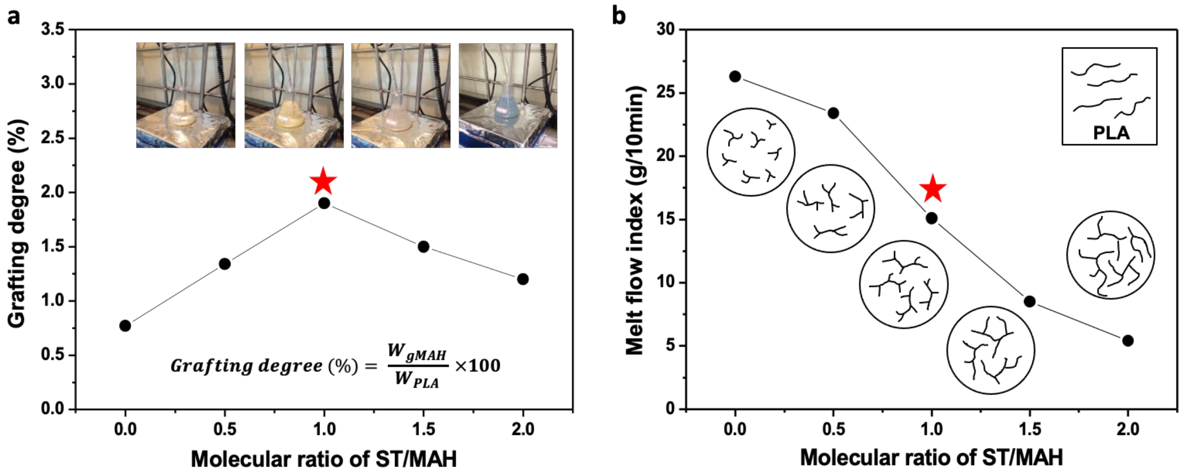

Figure S4. Determination of grafting degree and melt flow index (MFI). **a)** grafting degree and **b)** melt flow index (MFI) according to molecular ratio of ST/MAH. The grafting degree was obtained with a thymol blue indicator until a dark blue. Grafting polymerization leads to the formation of grafted branches and a decrease in molecular weight, which changes the melt flowability. Efficiency of grafting degree was dependent on the ST/MAH ratio. When the ratio is 1:1, the grafting efficiency reached its maximum level, and as the styrene content exceeded that of the MAH, the efficiency started to decrease due to the grafting competition between the MAH and styrene monomers caused by the imbalance in the ratio.

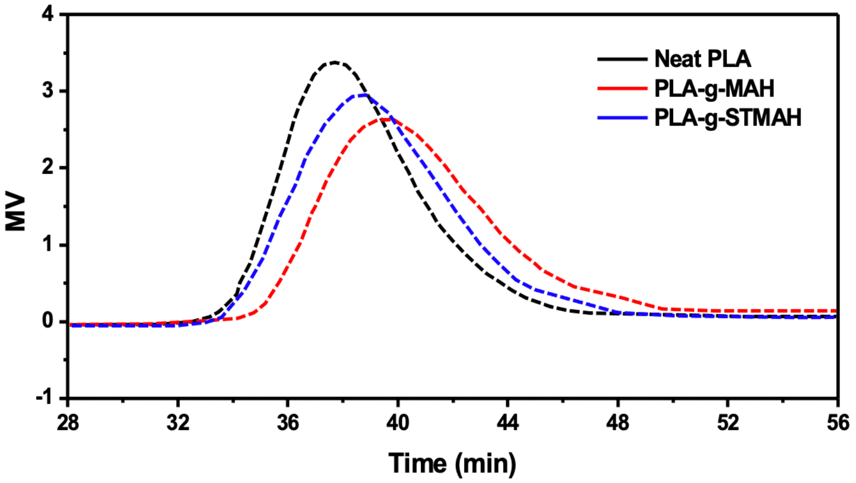

	Mn	Mw	PDI
Neat PLA	100,866	190,377	1.89
PLA-g-MAH	47,350	132,053	2.79
PLA-g-STMAH	80,184	174,231	2.17

Figure S5. GPC traces of Neat PLA, PLA-g-MAH and PLA-g-STMAH. The terms of Mw, Mn and PDI correspond to weight average molecular weight, number average molecular weight, and polydispersity index, respectively.

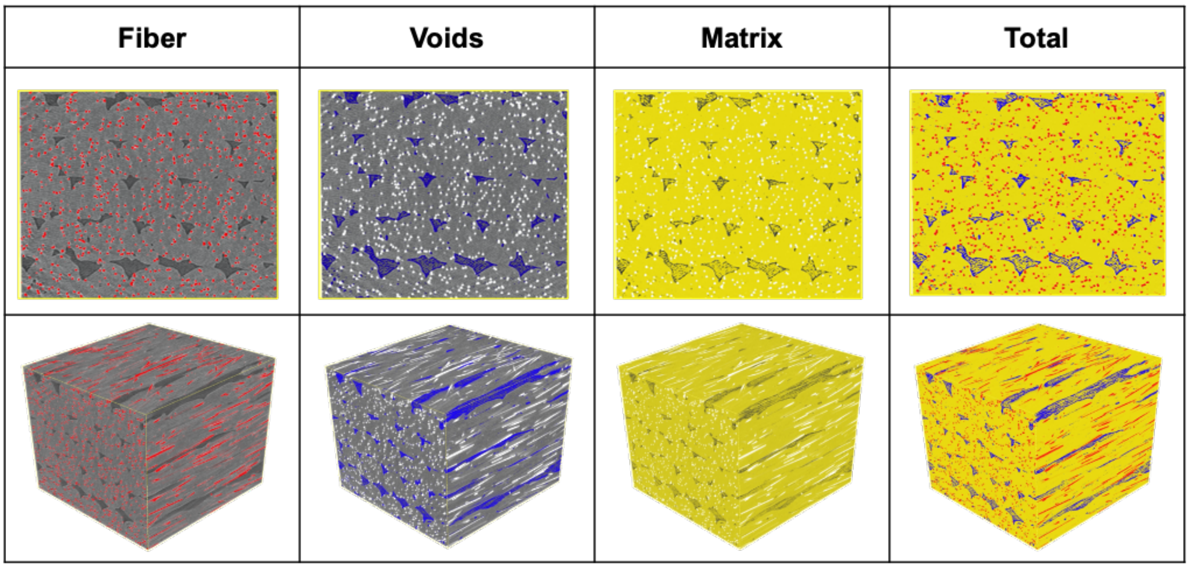

Figure S6. Segmentation images identifying all individual building elements, including fibers, voids and the matrix. Gray-scale images were classified into fibers, voids, and matrix according to the degree of grayness, and each of the grayness levels was matched with red, blue, and yellow colors.

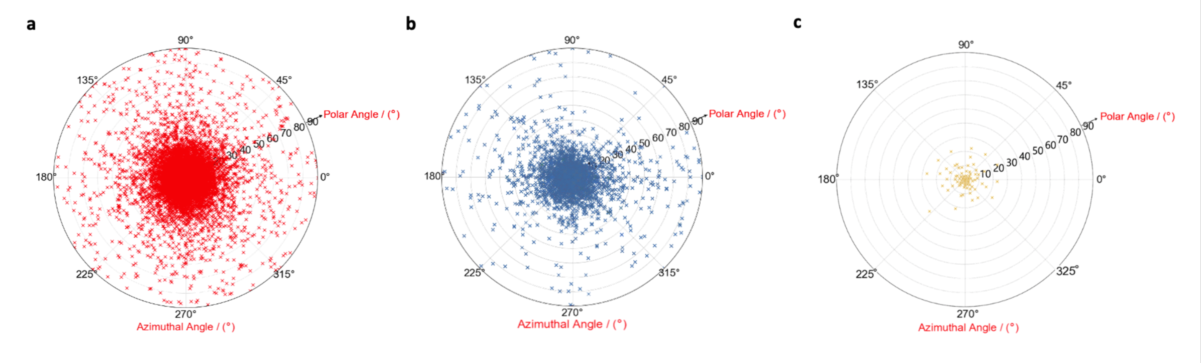

Figure S7. Orientation mapping images for three-dimensional distribution of fiber, voids and matrix. **a-c)** The spherical coordinate mapping images of orientation distribution of fiber (red), voids (blue) and matrix (yellow) showing a high concentration in one spot corresponding to a specific direction.

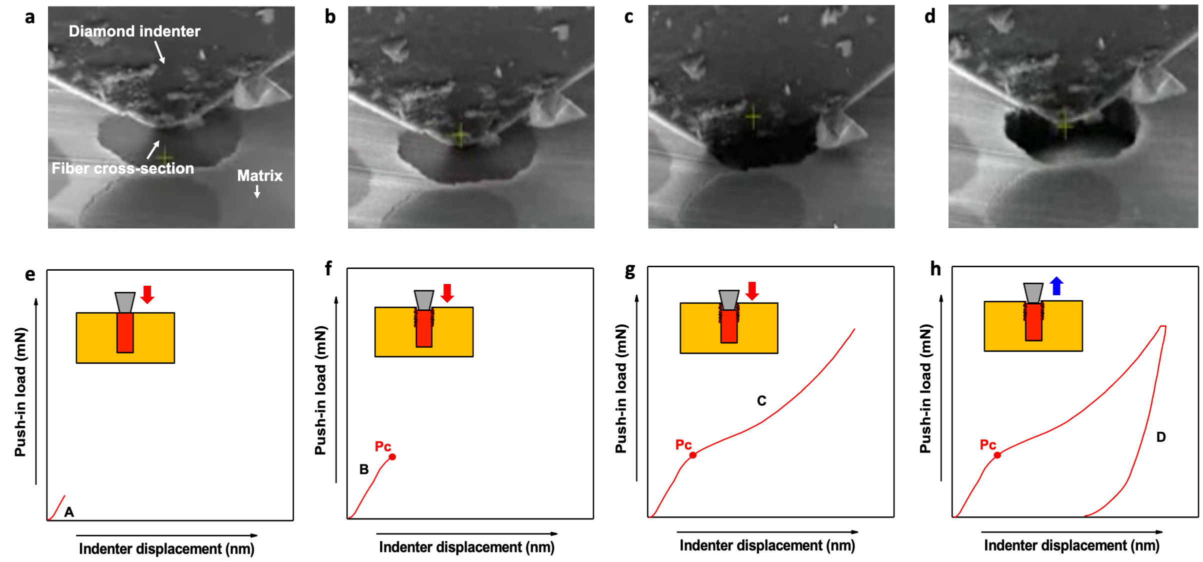

Figure S8. Nanoindentation push-in test for measuring interfacial shear stress. **a-d)** Sequential SEM images of in-situ nanoindentation experiments. **e-f)** Schematic illustration and indentation curves of interfacial crack propagation in nanoindentation push-in tests. The indentation curves were shown in 4 steps: the initial load segment (A) that represents incomplete contact between the indenter tip and the fibers, the linear region (B) where the fibers and matrix deform elastically under a load, the nonlinear region (C) where the slope starts to decrease following the onset of interfacial failure under load, and (D) the unloading curve.

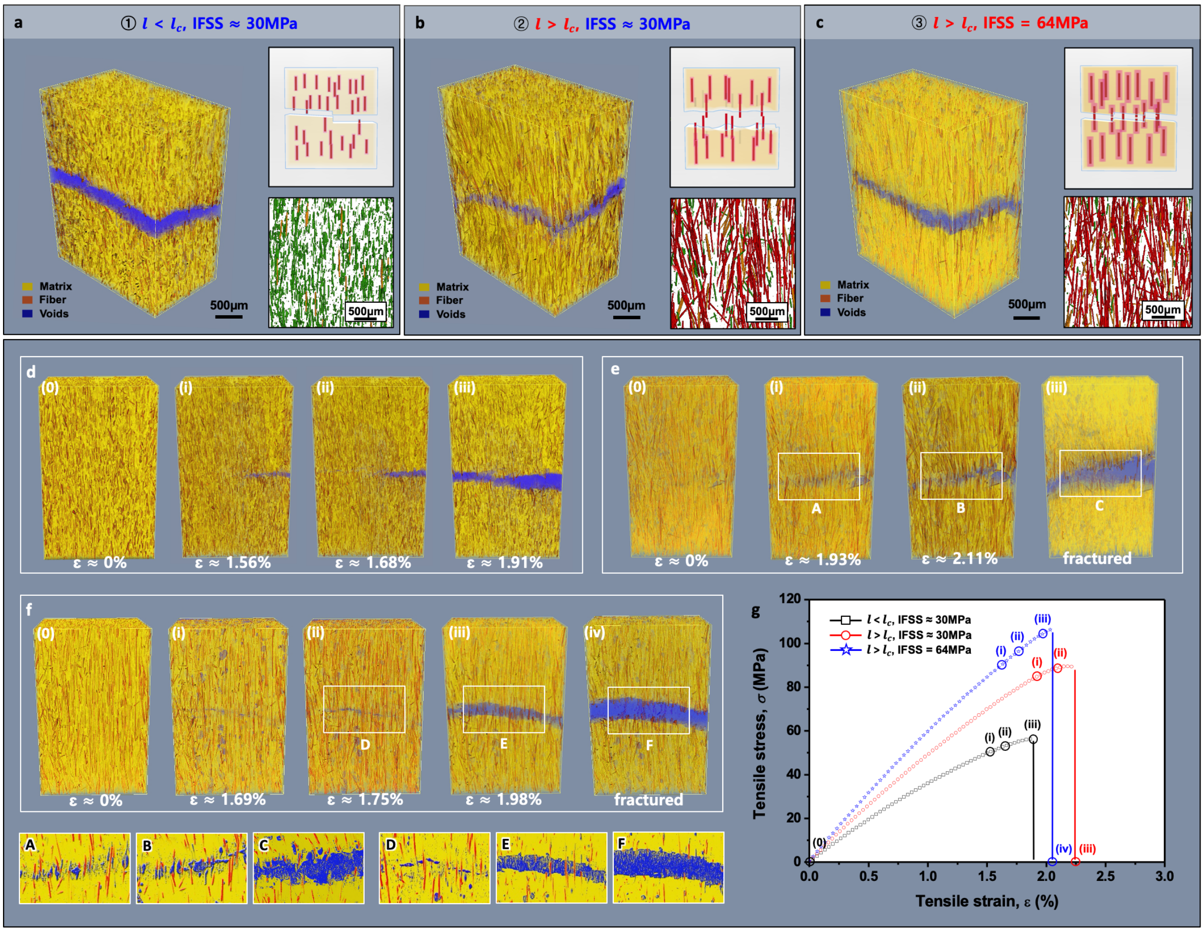

Figure S9. In-situ tomographic observation on 3D-printed SFRP composites under tensile loading condition. **a-c)** Representative 3D tomographic images showing final failures of 3D-printed single matrix BF/PLA/PLAgMAH composites. Various deformation and fracture modes based on fiber length and interfacial conditions; ① $l < l_c$, $l \approx 50$ μm, IFSS ≈ 30 MPa (**a**); ② $l > l_c$, $l \approx 700$ μm, IFSS ≈ 30 MPa (**b**); and ③ $l > l_c$, $l \approx 700$ μm, IFSS = 64 MPa (**c**). Color-coded tomography images showing fiber length distribution inside. Given as red-color fibers ($l > 600$ μm), yellow-color fibers (300 μm > l > 600 μm), and green-color fibers ($l < 300$ μm). **d-f)** Detailed illustration of the progressive failure process at each tensile loading sequence for 3D-printed single matrix BF/PLA/PLAgMAH composites. Progression of the deformation and fracture modes of specimens based on fiber length and interfacial conditions;

Submitted to ADVANCED SCIENCE Open Access

① $l < l_c$, $l \approx 50$ µm, IFSS ≈ 30 MPa (**d**); ② $l > l_c$, $l \approx 700$ µm, IFSS ≈ 30 MPa (**e**); and ③ $l > l_c$, $l \approx 700$ µm, IFSS = 64 MPa (**f**). 2D extended images A–F correspond to the marked regions for A–F in (**d-f**), respectively. **g)** Representative stress–strain curves of 3D-printed single matrix SFRP composites showing each tensile loading sequence at which a fractographic image was acquired with X-ray microscopy. Circle points represent the strains corresponding to the fractographic images in (**d-f**), respectively.
